# Combined antisaccade task and transcranial direct current stimulation to increase response inhibition in binge eating disorder

**DOI:** 10.1007/s00406-020-01164-5

**Published:** 2020-07-13

**Authors:** Sebastian M. Max, Christian Plewnia, Stephan Zipfel, Katrin E. Giel, Kathrin Schag

**Affiliations:** 1 Department of Psychiatry and Psychotherapy, Neurophysiology and Interventional Neuropsychiatry, University of Tübingen, Calwerstraße 14, 72076 Tübingen, Germany; 2 Department of Psychosomatic Medicine and Psychotherapy, University Hospital Tübingen, Osianderstraße 5, 72076 Tübingen, Germany; 3 Competence Center for Eating Disorders Tübingen (KOMET), Tübingen, Germany

**Keywords:** Antisaccade, Binge eating disorder, Cognitive control, Impulsivity, Response inhibition, Transcranial direct current stimulation

## Abstract

Binge eating disorder (BED) is associated with deficient response inhibition. Malfunctioning response inhibition is linked to hypoactivation of the dorsolateral prefrontal cortex (dlPFC), where excitability could be increased by anodal transcranial direct current stimulation (tDCS). Response inhibition can be assessed using an antisaccade task which requires supressing a dominant response (i.e. saccade) towards a newly appearing picture in the visual field. We performed a double-blind, randomised, placebo-controlled proof-of-concept-study in which we combined a food-modified antisaccade task with tDCS in people with BED. We expected task learning and modulatory tDCS effects. Sixteen people were allocated to a 1 mA condition, 15 people to a 2 mA condition. Each participant underwent the food-modified antisaccade task at three measurement points: baseline without stimulation, anodal verum and sham stimulation at the right dlPFC in a crossover design. The error rate and the latencies of correct antisaccades decreased over time. No tDCS effect on the error rate could be observed. Compared to sham stimulation, 2 mA tDCS decreased the latencies of correct antisaccades, whereas 1 mA tDCS increased it. Self-reported binge eating episodes were reduced in the 2 mA condition, while there was no change in the 1 mA condition. Participants demonstrated increased response inhibition capacities by a task learning effect concerning the error rate and latencies of correct antisaccades over time as well as a nonlinear tDCS effect represented by ameliorated latencies in the 2 mA and impaired latencies in the 1 mA condition. The reduction of binge eating episodes might indicate a transfer effect to everyday life. Given that the reduction in binge eating was observed before tDCS administration, this effect could not be the result of neuromodulation. Randomized clinical trials are needed to fully understand this reduction, and to explore the efficacy of a combined antisaccade and tDCS training for BED.

## Introduction

Since 2013, binge eating disorder (BED) is a distinct eating disorder diagnosis in the Fifth Edition of the Diagnostic and Statistical Manual of Mental Disorders (DMS-V). The estimated prevalence rate BED is with 1–4% higher than those of other eating disorders, and BED has a severe impact on individuals’ health and functioning level [1–3]. According to current treatment guidelines, the current treatment of choice is cognitive behavioural therapy (CBT). Unfortunately, only 50% of affected patients are fully remitted after CBT [4]. Thus, novel innovative treatments addressing the proposed underlying mechanisms of the disorder and targeting the brain directly, e.g. using neuromodulation techniques, could increase therapy benefit and remission rates in BED [5].

BED is characterized by recurrent binge eating episodes without inappropriate compensatory behaviours which is a core characteristic of bulimia nervosa. During binge eating episodes, an experienced loss of control is reported. This loss of control is closely linked to food-related impulsivity, i.e. binge eating could be understood as impulsive eating behaviour [6]. Two important factors that characterize impulsivity are, (1) increased sensitivity to rewarding stimuli, and (2) increased rash and spontaneous behaviour or decreased response inhibition [7, 8]. An executive function to overcome or correct impulsive behaviour in favour of another reaction is cognitive control [9, 10]. Thus, cognitive control and impulsivity are overlapping constructs concerning response inhibition. An experimental approach to operationalize response inhibition is the antisaccade paradigm, a task where an automatic and highly dominant reflectory gaze movement towards newly appearing stimuli has to be suppressed and corrected [11, 12]. In a modified antisaccade task with food vs. neutral control stimuli, it has been shown that participants with BED had more problems in inhibiting saccades towards food stimuli compared to matched normal weight and individuals with obesity who did not have an eating disorder [13]. Moreover, first attempts to use this response inhibition paradigm as a training programme for patients with BED in three sessions using only food stimuli delivered promising results that such a training might support patients to reduce binge eating episodes [14].

On a biopsychological level, response inhibition is associated with the dorsolateral prefrontal cortex (dlPFC) [15, 16]. Specifically, the right dlPFC is involved in tasks in which response inhibition is needed to overcome impulsive prepotent actions, thus resulting in goal-directed behaviour [17, 18]. There is evidence that impaired response inhibition in people with BED is more prominent, but not restricted to food-specific stimuli and may reflect a generally decreased capacity for response inhibition [19, 20]. In particular, reduced neural activity in the dlPFC and associated malfunctioning in response inhibition were observed in people with BED [21]. In the current study, we aimed to combine the food-modified antisaccade task with non-invasive brain stimulation to directly target the underlying neural networks.

An effective tool to modulate neural activity associated with response inhibition and food processing is transcranial direct current stimulation (tDCS) [5, 22, 23]. Through anodal tDCS, cortical excitability can be facilitated by application of a weak current (1–2 mA) to the scalp [24]. In a sample without mental disorders, it has already been demonstrated that response inhibition can be modulated by tDCS [25]. In the domain of BED, the evidence of tDCS is very scarce, as most studies investigate the effects of tDCS on food-related craving in non-eating-disordered samples or samples with other eating disorders [5, 22]. To our knowledge, only one study focused on food-related craving (and not response inhibition) in patients with BED, where food craving and food intake could be reduced due to anodal tDCS to the right dlPFC [26]. In people with bulimia nervosa, tDCS decreased the self-reported urge to binge eat and increased self-regulatory control [27]. This supports the hypothesis of a hypoactivated response inhibition network in people with BED. However, the participants in these studies did not execute disorder-relevant tasks during stimulation. A direct combination seems very promising to draw a direct link between neural activity and behavioural outcomes.

In the present randomized, placebo-controlled, double-blind proof-of-concept-study, the systematic effect on response inhibition of anodal tDCS to the right dlPFC combined with the food-modified antisaccade task [14] in a sample with diagnosed BED was investigated. Findings of this pilot study will be used to develop a suitable training programme for patients with BED, e.g. to determine optimal stimulation parameters, and to investigate the expected underlying mechanisms concerning food-related impulsivity and cognitive control. In addition to expected learning effects elicited by the repeated execution of the food-modified antisaccade task, anodal tDCS should additionally compensate for hypoactivity of the right dlPFC in people with BED, thus resulting in improved performance in this task in comparison with sham stimulation. After a baseline measure at T0, sham and verum stimulations were randomized in counterbalanced order across measurement point T1 and T2. Further, participants were allocated to either a group that received 1 mA tDCS or 2 mA tDCS. As different stimulation intensities yield different and possible nonlinear effects [28, 29], those two stimulation intensities were compared to explore the optimal intensity of stimulation.

Taken together, we expect that the patients improved in the food-modified antisaccade task over the three measurement points independently of the allocated condition, i.e. the order of sham/verum stimulation and its intensity (1 mA vs. 2 mA) (“learning effect”). Concerning tDCS, we additionally expect that the participants improved under verum stimulation compared to sham stimulation (“tDCS effect”). Improvement means that the participants executed less wrong antisaccades and faster correct antisaccades. Further, we compare the tDCS effects of the two groups with 1 mA vs. 2 mA stimulation. As a clinical measure, we explore, if and how the participants changed in the frequency of binge eating episodes over time. Last, we expect positive associations between self-reported trait impulsivity and food-related impulsivity with the performance in the food-modified antisaccade task.

## Methods

### Participants

Participants were adults with normal weight or overweight/obesity (BMI > 20 kg/m^2^) to exclude patients with restrictive eating patterns or subsyndromal anorexia nervosa and had to fulfil criteria for BED according to DSM-5 [30]. Exclusion criteria were: attention deficit hyperactivity disorder (ADHD), psychotic disorders, bipolar-I disorder, current alcohol or drug addiction, current suicidality, current pregnancy, current physical illness which influences weight or eating behaviour and unstable medication, neurological diseases, current medication with neuroleptics or benzodiazepines, current attendance to structured dieting programs, past bariatric operations, metallic implants in the head, eye diseases.

We included 31 participants in the first condition with 1 mA, but 15 were screening failures due to inappropriate self-reports concerning in- and exclusion criteria in a screening checklist that we realized at the diagnostics appointment (no BED: 8, ADHD: 4, bariatric surgery: 2, history of seizures: 1). Thus, they were discarded from the study and only those 16 patients who fulfilled in and exclusion criteria in the diagnostics session were randomised within the 1 mA condition to receive first sham and then verum tDCS or vice versa.

Based on the experiences from the 1 mA condition, we included a short screening on the phone concerning inclusion and exclusion criteria for the 2 mA condition. Thus, we included 18 participants in the 2 mA condition, where three subjects were excluded during diagnostics, (no BED: 2, ADHD: 1), so that 15 subjects were finally randomised within the 2 mA condition. The participants received a reimbursement for study participation. The study was approved by the ethics committee of the Medical Faculty Tübingen, Germany and all participants gave written informed consent.

### Study design

**Fig. 1:**
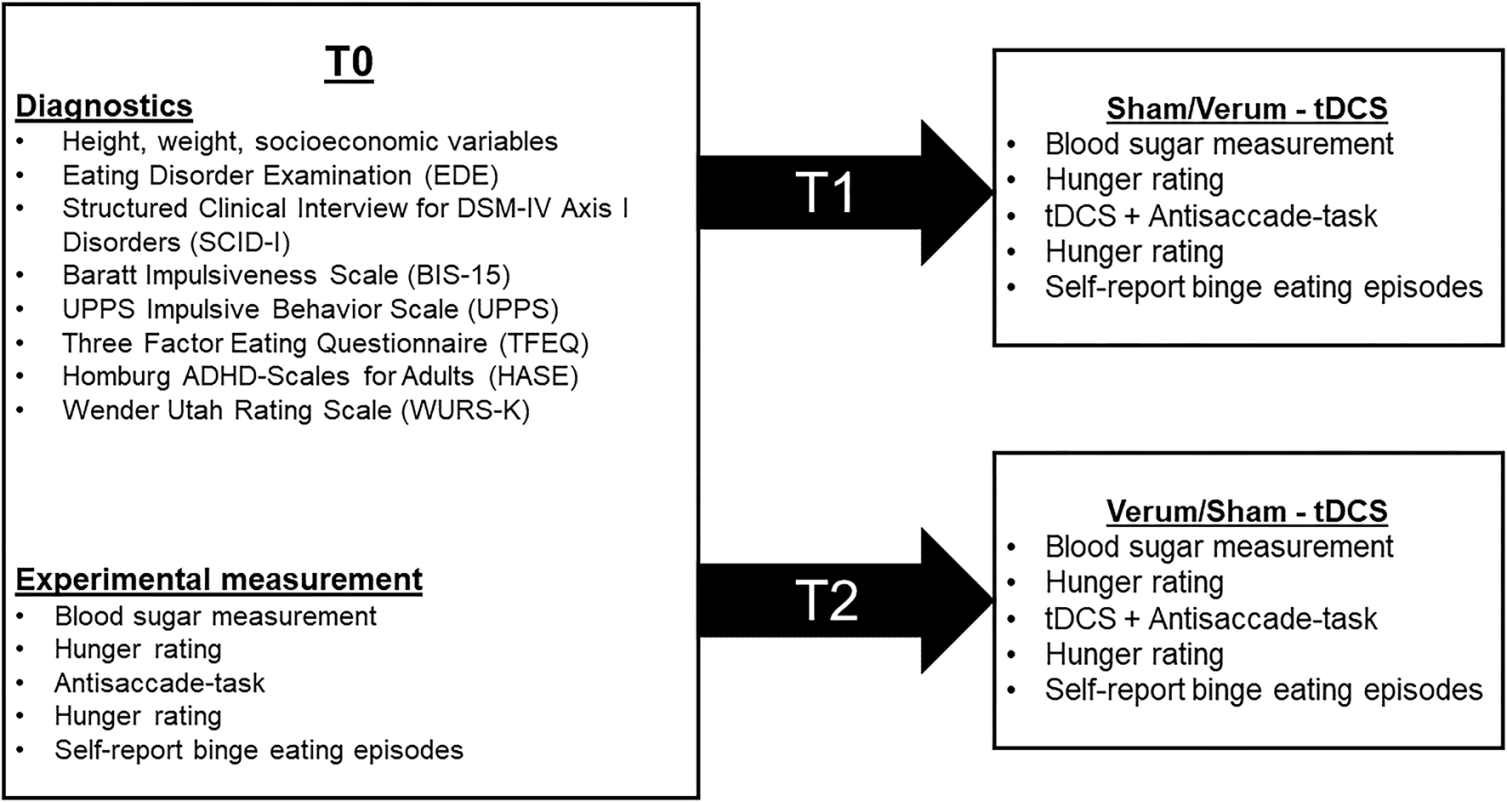
An overview of the three sessions (T0, T1 and T2) and the assessed data. The allocation to the two stimulation conditions (verum and sham stimulation) was randomized, counterbalanced and double-blind

This is a double-blind randomised placebo-controlled proof of concept study in a cross-over design (see Fig. 1). The intensity of the stimulation (1 mA vs. 2 mA) serves as a between-subject variable, as the participants were allocated to one specific stimulation intensity. The stimulation order (sham vs. verum) serves as a within-subject variable to which the participants got randomly assigned. If they received sham stimulation at the second study appointment (T1), they received verum stimulation at the third study appointment (T2) and vice versa. Counterbalancing of the stimulation order should minimize the influence of training effects.

### Food-modified antisaccade task

**Fig. 2:**
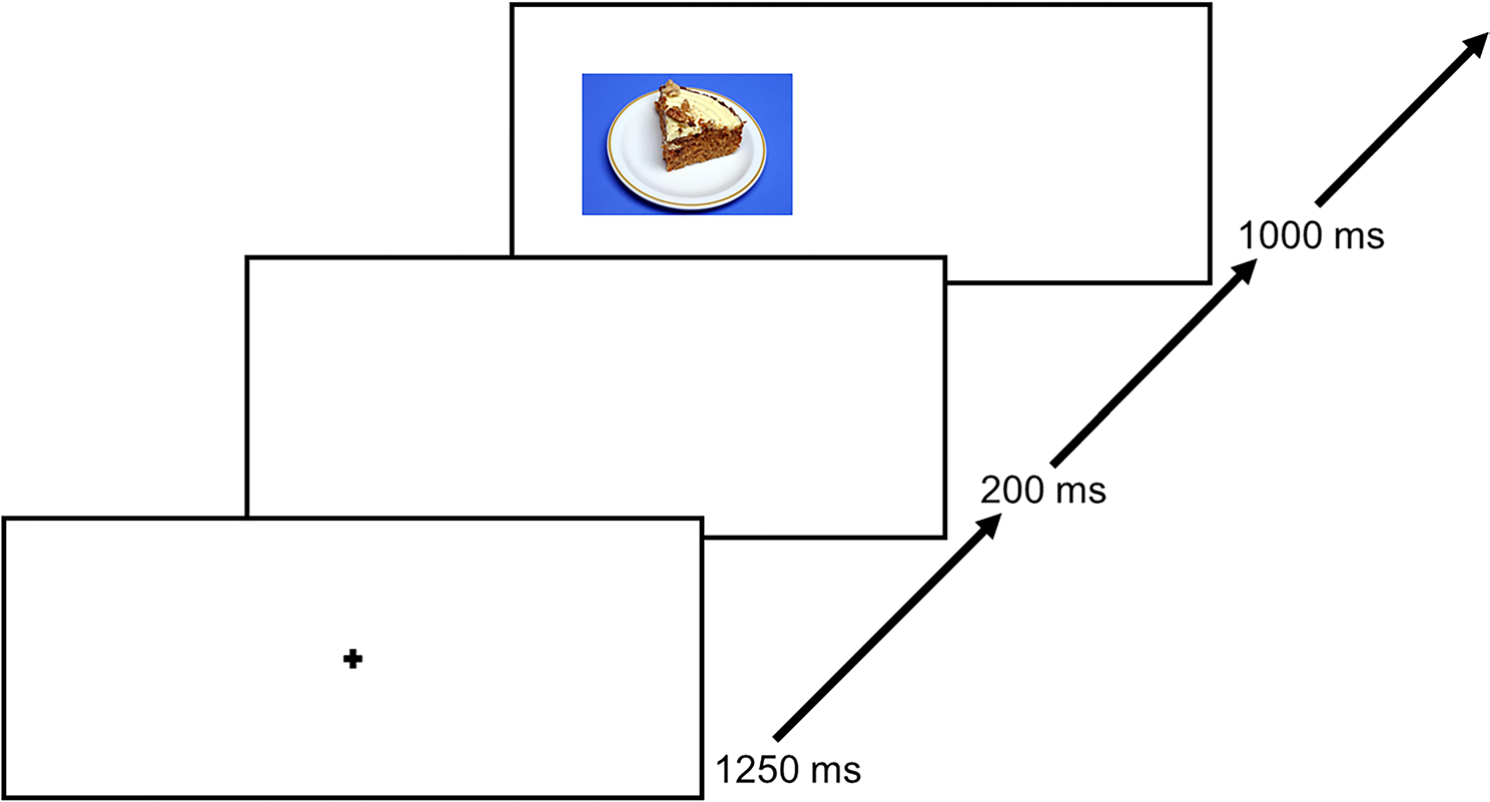
An exemplary trial course. The trial starts with a 1250 ms lasting fixation. After an ISI with 200 ms, a food picture was presented for 1000 ms. Thereafter, the next trial starts again with a fixation

In the food-modified antisaccade task, the participants were initially instructed to look at a fixation cross in the middle of the screen for 1250 ms in each trial as long as no picture was presented. After an interstimulus intervall (ISI) of 200 ms, a food picture was displayed randomly on the left or the right side of the screen for 1000 ms. As soon as the food picture was presented, the participants had to look as fast as possible on the opposite side of the screen. Each of the 40 food pictures was presented four times, counterbalanced on the left and the right sight on the screen, resulting in 160 trials. An exemplary trial is shown in Fig. 2.

### Stimuli and stimulus presentation

40 coloured high-caloric food stimuli with a resolution of 400 × 295 pixels served as stimulus material. The stimuli were pretested in other studies concerning response inhibition in BED [14, 31]. The stimulus material was displayed on 15.6-inch laptop screen with a resolution of 1280 × 1024 pixels.

### Apparatus

#### Eye tracking

Eye movements were recorded with SMI RED250mobile with a 250 Hz sampling rate, 0.4° gaze position accuracy, and iViewRed software. The mobile eye tracker, which was attached below the laptop screen, was placed 30 cm in front of the participant.

#### Eye movement data

Raw data were analysed with BeGaze 3.7 using the velocity-based default algorithms that define fixations and saccades. Data cleaning and composition of the output variables, i.e. trial classification (correct vs. error) and latencies were executed by MatLab R2017b. A trial was excluded from further analysis, if participants didn’t look at the fixation cross at the onset of the trial and if data were not recorded due to technical problems. Saccades starting below 80 ms and above 900 ms were considered premature/delayed, thus getting excluded from further analyses. As a marker of response inhibition, the error rate (ergo prosaccades to the food stimulus) and the latencies of correct antisaccades were used. Whereas the error rate reflects decreased response inhibition skills [12], the latency of correct antisaccades is indicating faster goal-directed behaviour and gives insight into the needed effort in inhibiting a reflexive prosaccade [32, 33].

#### Transcranial direct current stimulation (tDCS)

Transcranial direct current stimulation (tDCS) was delivered by two 5 × 7 cm electrodes which were both prepared with Ten20 conductive paste (Weaver and Company, Aurora, CO, USA). tDCS was administered over a 20 min period using a battery-driven, constant-current stimulator (DC-STIMULATOR MC, NeuroConn GmbH, Ilmenau, Germany). The cathode was placed extracephalic on the left deltoid muscle, the anode was placed over F4 according to the international 10–20 system of electrode placement [34]. Using a unipolar tDCS montage, we aimed to exclusively target the right dlPFC with anodal stimulation [35]. After a fade-in of 5 s, the current reached either 1 mA or 2 mA depending on the assigned group. After 20 min, the current was faded out within 5 s. For sham stimulation, the parameters of fade-in and fade-out were the same, but the current was only applied for 46 s. This is considered as a valid placebo-control as perceived sensations on the skin (e.g. tingling) usually fade out in the first 30 s of tDCS [36]. As putting a unique 5-digit code for each participant activates either real or sham stimulation, the experimenter was blinded to the randomisation condition (sham vs. verum).

### Questionnaires

#### Barratt impulsiveness scale (BIS-15)

The questionnaire assesses impulsivity as a personality trait operationalized by rapid, unplanned actions regardless of possible negative consequences. The questionnaire consists of three factors: non-planning, motor and attentional impulsivity. For the current study, only the total score was used which is a good marker of impulsivity and high internal consistency [37]. A higher total score indicates higher general impulsivity.

#### UPPS impulsive behaviour scale (UPPS)

The questionnaire measures and conceptualizes impulsivity with four facets: urgency, lack of premeditation, lack of perseverance, and sensation seeking. Urgency captures the tendency to undergo strong impulses mainly under the influence of negative affect. Premediation measures the tendency to act deliberately and consciously. Perseverance describes the ability to stay focussed on a boring or difficult task. Sensation seeking comprises the tendency to enjoy risky and exciting activities and openness for new experiences [38]. A higher score of the different subscales indicates either higher urgency, lower premeditation, lower perseverance or higher sensation seeking.

#### Three-factor eating questionnaire (TFEQ)

The questionnaire consists of three scales to conceptualize eating behaviour by behavioural, cognitive, and affective components. Restraint comprises strategic dieting behaviour, the ability of self-regulation and avoidance of fattening foods. Disinhibition refers to habitual, emotional, and situational susceptibility. The last component is hunger which comprises internal and external processing for hunger cues [39]. A higher score of the subscale indicates a higher extent of the eating behaviour component.

### Procedure

An overview of the study appointments is depicted in Fig. 1. Between each of the three study appointments, there was a minimum temporal distance of 1 week to control for carry-over effects as this seems to be a sufficient wash-out period [40–42]. To control for circadian effects, participants came at the same time in the late afternoon/early evening for each study appointment. To hold homeostatic effects constant, the participants were instructed to fast at least 4 h before they arrived at the laboratory to be in a moderately hungry state while completing the tasks, as hunger increases food-related attentional and motivational processes [43]. Blood sugar levels were assessed as well as hunger levels at visual analogue scales ranging from 0 cm (not hungry) to 10 cm (extremely hungry).

At the diagnostics appointment at T0, height, weight and socioeconomic variables were assessed. To check in and exclusion criteria, we executed two structured interviews for current eating disorder diagnoses (EDE, [44] and SCID-I, [45]). To assess BED according to DSM-5, we slightly modified the German version of the EDE instead of DSM-IV in that way that an average of one binge eating episode per week within 3 months was necessary to diagnose BED instead of an average of two binge eating episodes per week within 6 months. To characterise the sample, participants filled out standardized questionnaires concerning impulsivity (BIS-15 [37]; UPPS Impulsive Behaviour Scale [38]), eating behaviour (TFEQ [39]) and ADHD (HASE [46]; WURS-K [47]) (see above). At the same appointment, the experimental baseline measurement with the food-modified antisaccade task was conducted. Afterwards, a self-report about the frequency of binge eating episodes in the last 7 days was filled out.

The following two experimental measurements at T1 and T2 were similar to T0 with the exception that additionally tDCS (verum/ sham) was applied. At T2, participants had to guess in a blinding check which session verum stimulation was applied and rated frequency as well as intensity of adverse events on a scale ranging from 1 (“not at all”) to 5 (“extremely”).

### Data analysis and statistics

#### Behavioural data analysis

All statistical inferences were conducted on a significance level of 95%. The sample was compared in the two study conditions (1 mA vs. 2 mA) with t tests or Mann–Whitney U tests, if data were not normally distributed, and Chi square tests for binary data. Manipulation and blinding checks were executed with t tests.

Concerning eye tracking data, participants with less than 25 trials at T0 due to bad data quality (i.e. recording problems) were totally excluded (*N* = 4), so that *N* = 15 were analysed in the condition 1 mA and *N* = 12 in the condition 2 mA. To account for different datapoint contributions to the analysis, mixed models were calculated by the *lme4*-package of R [48]. To analyse the error rate, generalized linear mixed models were performed which return the logit of an error and transforms it into a probability. To analyse the latencies of correct antisaccades, linear mixed models were performed which return a numeric estimate for each included factor level. Two more participants in the condition 2 mA had to be excluded in the analysis on latencies of correct antisaccades, as they did not perform any correct saccades at T0 to calculate a valid baseline covariate. To investigate stimulation-independent learning effects, the fixed effect of session (T0, T1, and T2) was tested in log-likelihood tests. Incorrect executed trials (see above) were excluded from the analysis, so that concerning the error rate, 7922 trials (61.13%), and concerning the latencies of correct antisaccades, 5013 trials (41.78%) could be analysed. To investigate stimulation-dependent effects, two fixed effects were tested: stimulation (verum vs. sham), intensity (1 mA vs. 2 mA). Only the trials of the two stimulation sessions (T1, T2) were analysed and an individual baseline performance was also included as a fixed effect to serve as a correction factor for learning effects. Concerning the error rate, 5146 trials (59.56%), and concerning the latencies of correct antisaccades, 3428 trials (42.85%) could be analysed.

The self-reported frequency of binge eating episodes in the last 7 days was analysed consistently with a linear mixed model including the session (T0, T1, T2) and the stimulation intensity (1 mA vs. 2 mA) as fixed effects.

Concerning all variables, post hoc contrasts within fixed effects were tested with the method of least-squares means [49] by the *lsmeans* package of R and adjusted by Tukey method. Cohen’s *d* was used as a standardized effect-size measure [49]. As the eye tracking data were not normally distributed, Spearman’s correlations between the BIS-15, UPPS and TFEQ and the eye tracking performance at T0 were executed.

## Results

### Sample characteristics

The sample characteristics are described in Table 1. The samples in the two study conditions did not differ from each other concerning the outcomes presented in Table 1 and additionally concerning blood glucose level, education, marital status and frequency of comorbid mental disorders.

**Table 1:** Sample characteristics at baseline

	1 mA (*N* = 15)	2 mA (*N* = 12)	*p*
Age	35.7 (13.0)	40.6 (15.6)	0.52
Sex	1 m, 14 f	3 m, 9 f	0.18
BMI (kg/m^2^)	32.1 (10.9)	33.8 (9.6)	0.67
Binge eating days in the last 4 weeks according to EDE	11.3 (5.7)	12.8 (7.8)	0.87
EDE total score	1.8 (.7)	2.0 (.9)	0.61
TFEQ disinhibition	11.3 (3.2)	11.6 (2.6)	0.78
TFEQ feelings of hunger	8.5 (2.7)	10.3 (2.2)	0.08
TFEQ cognitive restraint	7.9 (4.0)	6.1 (3.2)	0.19
BIS-15 total score	30.1 (7.1)	27.6 (2.6)	0.30
UPPS urgency	31.5 (5.8)	29.3 (4.6)	0.29
UPPS premediation	24.3 (4.3)	24.3 (3.2)	0.98
UPPS lack of perseverance	19.9(2.9)	22.3 (3.6)	0.07
UPPS sensation seeking	30.9 (8.3)	29.8 (9.3)	0.73

*BIS-15* Baratt Impulsiveness Scale *EDE* eating disorder examination; *f* female; *m* male; *TFEQ* three-factor eating questionnaire; *UPPS* UPPS Impulsive Behaviour Scale

### Manipulation and blinding check

Blood sugar levels across all participants and measurement points were on average at 100.0 mmol/l (SD = 19.6) and rated hunger levels at 54.8 (SD = 19.1), which speaks for a moderately hungry state before each experimental measure as expected. The probability of the correctly guessed verum stimulation session was at chance level (55.56%), *t*(26) = 0.57, *p* = 0.574 and did not differ between groups, (1 mA = 60%; 2 mA = 50%), *t*(25) = 0.50, *p* = *0.620*. Thus, a blinding of the participants overall can be assumed. None of the participants reported severe adverse events, but some light and already well-known adverse events were reported: 22 (81.5%) participants reported tingling of the electrodes (*M* = 2.52), 8 (29.6%) tingling on the head (*M* = 1.41), 12 (44.4%) itching (*M* = 1.78), 8 (29.6%) exhaustion (*M* = 1.33), 6 (22.2%) headache (*M* = 1.26), 2 (7.3%) others (*M* = 1.19) and no one reported sickness.

### Error rate

#### Learning effects

**Fig. 3:**
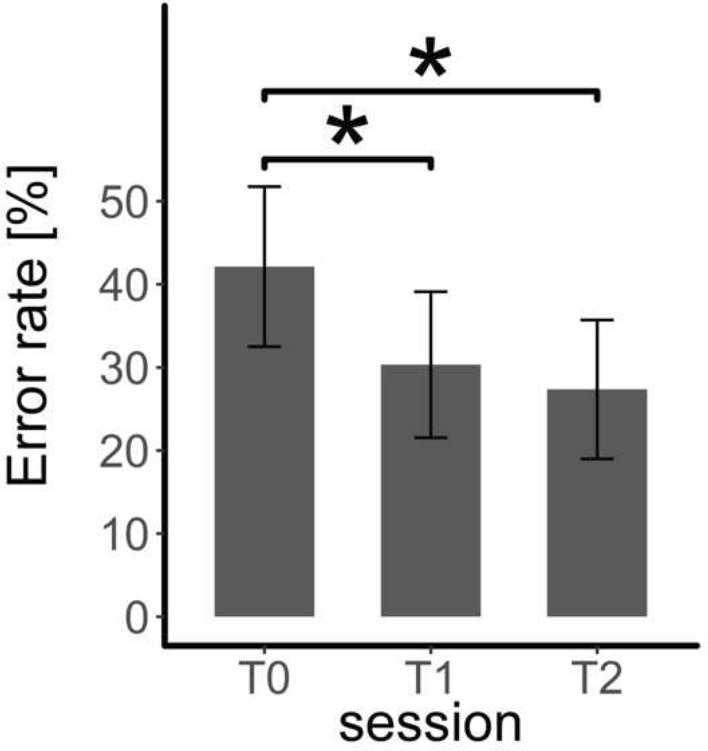
The error rate (mean, standard error) at each study appointment (T0, T1 and T2). * *p* < .05

Session as a fixed effect led to a significantly better model than the random intercept-only model, *χ*^2^(3) = 85.97, *p* < 0.001, *R*^2^ = 0.55. As illustrated in Fig. 3, post hoc tests revealed a significantly lower error rate at T1 compared to T0 (*β* = − 0.51, SE = 0.08, *z* = 6.83, *p* < 0.001) and at T2 compared to T0 (*β* = − 0.66, SE = 0.08, *z* = 8.54, *p* < 0.001). T1 and T2 did not differ (*β* = − 0.14, SE = 0.08, *z* = 1.86, *p* = 0.151).

#### tDCS effects

**Fig. 4:**
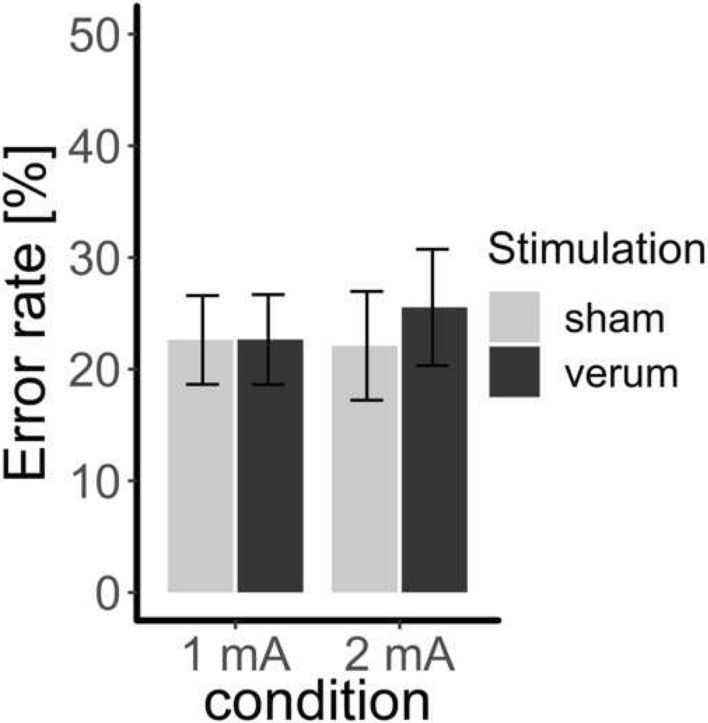
The error rate (mean, standard error) depending on condition (1 mA vs. 2 mA) and stimulation (verum vs. sham) * *p* < .05

Stimulation (sham vs. verum) did not have an effect on the error rate, *χ*^2^(2) = 5.92, *p* = 0.052, R^2^ = 0.56 (see Fig. 4). Intensity as a fixed effect led to a significantly better model *χ*^2^(2) = 6.83, *p* = 0.033, R^2^ = 0.56, but post hoc tests did not reveal a significant difference between 1 mA vs. 2 mA (*β* = 0.93, SE = 0.77, *z* = 1.20, *p* = 0.231). Also, within each intensity condition, no significant contrast between sham and verum stimulation emerged (*p* > 0.05).

### Latencies of correct antisaccades

#### Learning effects

**Fig. 5:**
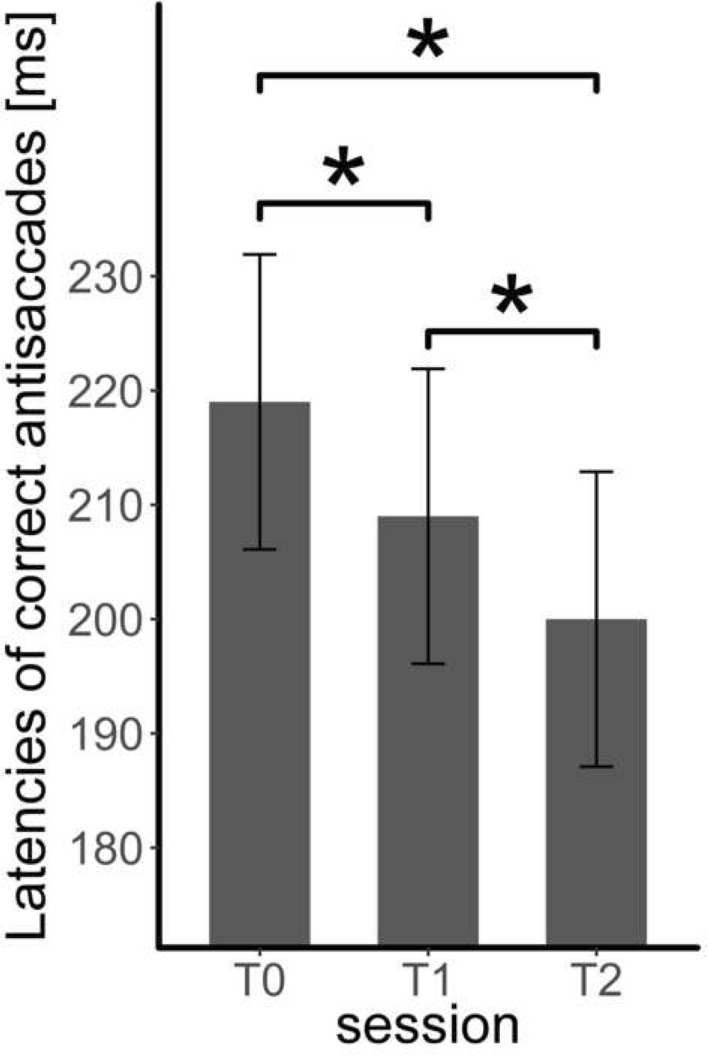
The latencies of correct antisaccades (mean, standard error) at each study appointment (T0, T1 and T2) * *p* < .05

Session as a fixed effect led to a significantly better model, *χ*^2^(3) = 168.14, *p* < 0.001, *R*^2^ = 0.60 (see Fig. 5). Post hoc tests reveal faster latencies of correct antisaccades at T1 vs. T0 (*β* = 9.78 ms, SE = 1.88, *z* = 5.20, *p* < 0.001), at T2 vs. T0 (*β* = 19.25 ms, SE = 1.85, *z* = 10.39, *p* < 0.001) and at T2 vs. T1 (*β* = 9.48 ms, SE = 1.83, *z* = 5.16, *p* < 0.001).

#### tDCS effects

**Fig. 6:**
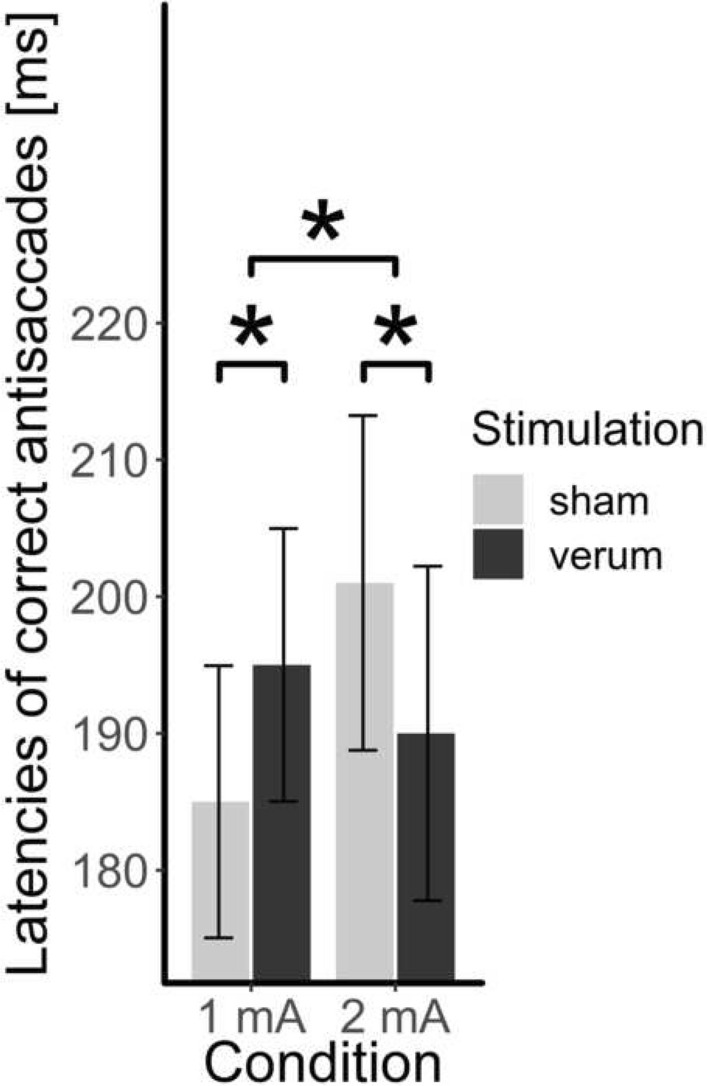
The latencies of correct antisaccades (mean, standard error) depending on condition (1 mA vs. 2 mA) and stimulation (verum vs. sham) * *p* < .05

The interaction between the fixed effect of stimulation and intensity led to the best model, *χ*^2^(1) = 37.50, *p* < 0.001, *R*^2^ = 0.49 (Fig. 6). At the 1 mA condition, post hoc tests revealed significantly slower latencies of correct antisaccades under verum stimulation than sham stimulation (*β* = − 10.70 ms, SE = 2.27, *z* = − 4.71, *p* < 0.001, *d* = 0.22). With 2 mA stimulation, latencies of correct antisaccades were significantly faster under verum stimulation than sham stimulation (*β* = 11.29 ms, SE = 2.77, *z* = 4.08, *p* < 0.001, *d* = 0.23).

### Change of binge eating episodes

**Fig. 7:**
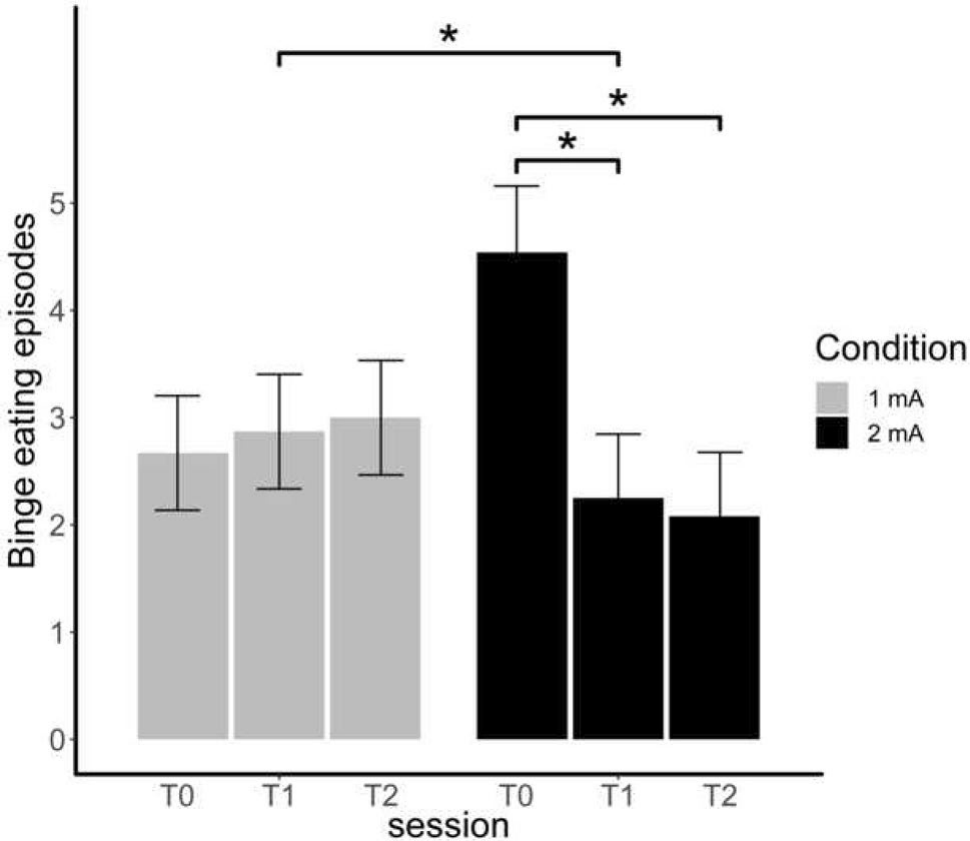
Self-reported frequency of binge eating episodes in the last 7 days (mean, standard error) depending on condition (1 mA vs. 2 mA) and session (T0, T1 and T2) * *p* < .05

The interaction between the fixed effect of session and intensity (Fig. 7) led to the best model (*χ*^2^(2) = 10.89, *p* = 0.004, *R*^2^ = 0.23). The frequency of binge eating episodes decreased at the 2 mA condition (*β* = 2.46, SE = 0.69, *z* = 3.56, *p* = 0.009, *d* = 1.56), representing a strong effect, whereas it did not change significantly at the 1 mA condition (*β* = − 0.33, SE = 0.60, *z* = -0.56, *p* = 0.993, *d* = 0.21).

### Correlational analyses

A significant positive correlation was found between the BIS-15 total score and the error rate (*r*_*s*_ = 0.60, *S* = 1182.10, *p* = 0.001), whereas no significant correlation was found with the latencies of correct antisaccades (*r*_*s*_ = 0.34, *S* = 1727.60, *p* = 0.101). A significant positive correlation was found between the UPPS urgency scale and the latencies of correct antisaccades (*r*_*s*_ = 0.46, *S* = 1391.80, *p* = 0.019), whereas no significant correlation was found with the error rate (*r*_*s*_ = 0.36, *S* = 2106.80, *p* = 0.068). The other UPPS subscales and the TFEQ subscales did not correlate significantly with the eye tracking variables.

## Discussion

In this double-blind, randomised, placebo-controlled proof of concept study, we investigated learning effects on a food-modified antisaccade task addressing response inhibition, and the effects of anodal 1 mA and 2 mA tDCS to the right dlPFC in a sample with BED. Overall, we could show improved response inhibition towards food stimuli as well as reduced binge eating frequency over time in the 2 mA condition. In line with our hypothesis about learning effects, patients improved over the three measurement points concerning error rate and latencies of correct antisaccades. Concerning our hypothesis about tDCS effects, the stimulation did not affect error rates, but the group that received 2 mA stimulation improved with faster latencies of correct antisaccades compared to sham stimulation, whereas the group that received 1 mA stimulation showed slower latencies. Moreover, only in the group that received 2 mA, a significant decrease of self-reported binge eating episodes over time could be observed, whereas no change was evident in the group that received 1 mA. Last, few, but strong positive associations between self-reported trait impulsivity with the performance in the food-modified antisaccade task could be found, i.e. BIS-15 was associated with an increased error rate and urgency correlated with longer latencies of correct antisaccades.

In more detail, a clear learning effect in the food-modified antisaccade task throughout the three measurement points could be observed. The error rate and the latencies of correct antisaccades decreased, indicating ameliorated response inhibition and goal-directed behaviour within three sessions. This is in line with previous training studies, where the error rates also decreased throughout the training [14]. This strengthens the assumption that underlying cognitive impairments in patients with BED can be modified by a repeated execution of a disorder-related task. Moreover, especially response inhibition tasks seem to be a useful basis for computer-assisted training programs in BED.

Concerning tDCS, no effect on the error rate of antisaccades could be observed. This is consistent with the notion that tDCS mainly influences the reaction times, not the accuracy of a response [50]. Accordingly, we observed faster latencies of correct antisaccades in the 2 mA condition which indicates that with this stimulation intensity, less effort is needed to execute the task. In other cognitive domains, it could already be demonstrated that a higher intensity is not inevitably accompanied by a better performance [29, 51], but in the current study, a lower intensity of tDCS (1 mA) even led to a worse performance. In the domain of BED, the positive effect of 2 mA tDCS is concordant with the study of Burgess et al. [26] who demonstrated enhanced cognitive control in patients with BED after 2 mA tDCS using bipolar montage. Even if Burgess et al. [26] applied anodal stimulation to the right dlPFC simultaneously to cathodal stimulation to the left dlPFC which potentially impacts behaviour differently, a central role of the right dlPFC in BED can be assumed. The effect of 2 mA tDCS on the latency of correct antisaccades supports the idea of compensating the hypoactivated right dlPFC in patients with BED while doing the antisaccade task that demands response inhibition in a great extent. This provides support for the idea that underlying neural networks in response inhibition are altered in patients with BED and that this biopsychological marker can be targeted by tDCS. By application of 2 mA tDCS, the cortical excitability of the right dlPFC can be increased and, therefore, a significant improvement of response inhibition can be achieved. The possibility to modulate hypoactivated neural networks by 2 mA tDCS offers new options in multimodal treatment of BED.

Going beyond the experimental outcomes, a significant reduction of self-reported binge eating episodes over the measurement points emerged in the 2 mA condition contrary to the 1 mA condition. It is puzzling that the strongest decrease of binge eating episodes happened from T0 to T1 as we did not administer tDCS at T0. It could be that the learning effect from the food-modified antisaccade task on response inhibition was transferred to the psychopathology of patients with BED in everyday life. But in this case, we would expect ameliorations in both, the 1 mA and 2 mA condition. Another interpretation might be that this effect was due to the participation in the study itself, but this does also not explain why we found this effect only in the 2 mA and not in the 1 mA condition. In another pilot study from Giel et al. [14], binge eating frequency was also reduced after the antisaccade training, though the control group reduced binge eating frequency as well. Shafran et al. [52] could show in patients with eating disorders that decreased eating disorder pathology after CBT was accompanied by a decreased attentional bias in a laboratory task. Thus, although there is only limited and preliminary evidence, our results and the results from the two other studies might indicate that there could be a direct link between neurocognitive impairments like response inhibition and psychopathology like binge eating in BED. Thus, a coupling of non-invasive brain stimulation with a response inhibition training might be efficacious to reduce clinical outcomes as well.

One strength of this pilot study is that we assessed a representative sample of patients with BED who showed increased feelings of hunger and disinhibition while eating (TFEQ) compared with the general population [53]. This is in line with our theoretical model of increased impulsive eating behaviour in BED [6]. Concerning methodological aspects, to our knowledge, this is the first study combining neuromodulation techniques simultaneously with a behavioural task, where to date only one study concerning patients with BED is still ongoing [54]. The study was feasible and highly accepted, as the blinding worked, the participants complied with the 4-h fast, they reported only slight adverse events which are already well known and no one terminated the study due to discomfort, thus supporting the usage of tDCS in BED patients.

Nonetheless, some limitations should be addressed. The sustainability of the learning effect beyond the three sessions couldn’t be evaluated as there was no follow-up assessment scheduled. Such a follow-up may be investigated in a systematic training study. Eating disorder pathology and trait impulsivity were not increased in the sample which might be a result of the excluded patients with increased scores in the ADHD scales. However, the correlations between impulsivity self-reports and eye tracking outcomes indicate that facets of trait impulsivity have been operationalized with the food-modified version of the antisaccade task. Impaired response inhibition in people with BED may be generalised rather than food specific [19]. Another limitation concerning the blinding of the participants to the stimulation is that we did not ask how confident they were about the session in which they got verum or sham stimulation though it is known, that expectations of receiving tDCS can significantly impact tDCS outcomes [55]. However, in the present study, we observed a nonlinear tDCS effect where participants in the 1 mA condition even got worse under verum stimulation. This is unlikely for expectation effects. Moreover, we observed a 55% rate of right guesses which is at chance level and participants in the 1 mA and 2 mA condition did not differ from each other. Unfortunately, we had to exclude a few participants and a considerable proportion of trials from data analyses to keep data quality high. However, with at least 3400 trials in each analysis and a robust statistical approach which takes interindividual variation into account, we could still analyse a huge and representative dataset.

## Conclusion

In sum, this pilot study was carried out to combine tDCS with the food-modified antisaccade task to directly target food-related response inhibition as an intersection of impulsivity and cognitive control. The results suggest the modifiability of cognitive and biopsychological mechanisms in patients with BED and indicate that such a training programme with 2 mA stimulation of the right dlPFC might be useful for patients with BED concerning response inhibition. Based on these results, we will take the next step and develop such a training programme and explore its efficacy concerning clinical outcomes in a randomized, placebo-controlled, double-blind clinical trial that might enhance current CBT and decrease symptomatology in BED.

## Data Availability

Data can be obtained on request to the author.
